# Ischemic Mitral Valve Regurgitation in Patients Undergoing Coronary Artery Bypass Grafting—Early and Late-Term Outcomes of Surgical Treatment

**DOI:** 10.3390/jcm14144855

**Published:** 2025-07-09

**Authors:** Paweł Walerowicz, Mirosław Brykczyński, Aleksandra Szylińska, Jerzy Pacholewicz

**Affiliations:** 1 Department of Cardiac Surgery, Pomeranian Medical University, 70-111 Szczecin, Poland; pawel.walerowicz@pum.edu.pl (P.W.); jerzy.pacholewicz@pum.edu.pl (J.P.); 2 Department of Cardiac Surgery and Interventional Cardiology, Faculty of Medicine and Medical Sciences, University of Zielona Gora, 65-046 Zielona Gora, Poland; mirobryk@gmail.com

**Keywords:** cardiac surgery, mitral regurgitation, CABG, coronary artery bypass grafting

## Abstract

**Background:** Coronary heart disease (CHD) remains the most prevalent pathology within the circulatory system. Among its chronic complications, ischemic mitral valve regurgitation (IMR) is observed in approximately 15% of patients with sustained myocardial ischemia. The presence of this complex valvular defect significantly increases both overall mortality and the incidence of adverse cardiovascular events. Notably, the presence of moderate to severe mitral regurgitation in patients undergoing surgical revascularization has been shown to double the risk of death. Despite the well-established etiology of IMR, data regarding the efficacy of surgical interventions and the determinants of postoperative outcomes remain inconclusive. **Methods:** The objective of the present study was to evaluate both early and long-term outcomes of surgical treatment of mitral regurgitation in patients undergoing coronary artery bypass grafting (CABG) due to ischemic heart disease. Particular attention was given to the influence of the severity of regurgitation, left ventricular ejection fraction (LVEF), and the dimensions of the left atrium (LA) and left ventricle (LV) on the postoperative prognosis. An additional aim was to identify preoperative risk factors associated with increased postoperative mortality and morbidity. A retrospective analysis was conducted on 421 patients diagnosed with ischemic mitral regurgitation who underwent concomitant mitral valve surgery and CABG. Exclusion criteria included emergent and urgent procedures as well as non-ischemic etiologies of mitral valve dysfunction. **Results:** The study cohort comprised 34.9% women and 65.1% men, with the mean age of 65.7 years (±7.57). A substantial proportion (76.7%) of patients were aged over 60 years. More than half (51.5%) presented with severe heart failure symptoms, classified as NYHA class III or IV, while over 70% were categorized as CCS class II or III. Among the surgical procedures performed, 344 patients underwent mitral valve repair, and 77 patients required mitral valve replacement. Additionally, 119 individuals underwent concomitant tricuspid valve repair. Short-term survival was significantly affected by the presence of hypertension, prior cerebrovascular events, and chronic kidney disease. In contrast, hypertension and chronic obstructive pulmonary disease were identified as significant predictors of adverse late-term outcomes. **Conclusions:** Interestingly, neither the preoperative severity of mitral regurgitation nor the echocardiographic measurements of LA and LV dimensions were found to significantly influence surgical outcomes. The perioperative risk, as assessed by the EuroSCORE II (average score: 10.0%), corresponded closely with observed mortality rates following mitral valve repair (9.9%) and replacement (10.4%). Notably, the need for concomitant tricuspid valve surgery was associated with an elevated mortality rate (12.4%). Furthermore, the preoperative echocardiographic evaluation of LA regurgitation severity, as well as LA and LV dimensions, did not exhibit a statistically significant impact on either early or long-term surgical outcomes. However, a reduced LVEF was correlated with increased long-term mortality. The presence of advanced clinical symptoms and the necessity for tricuspid valve repair were independently associated with a poorer late-term prognosis. Importantly, the annual mortality rate observed in the late-term follow-up of patients who underwent surgical treatment of ischemic mitral regurgitation was lower than rates reported in the literature for patients managed conservatively. The EuroSCORE II scale proved to be a reliable and precise tool in predicting surgical risk and outcomes in this patient population.

## 1. Introduction

Ischemic mitral regurgitation (IMR) is one of the most complex complications of ischemic heart disease. It is most commonly classified as type IIIb, where regurgitation is caused by a restrictive limitation of the posterior mitral leaflet’s motion despite normal leaflet morphology [1].

Unlike mitral regurgitation of other etiologies, IMR is a disease of the entire heart, as valve regurgitation occurs due to ischemia-induced remodeling [2,3,4]. Two primary mechanisms underlie the pathogenesis of changes in the left ventricle and subsequent dysfunction of the mitral apparatus.

The first mechanism involves ischemia-related papillary muscle dysfunction. The second is left ventricular dilation accompanied by impaired contractility [5]. Ischemia of the left ventricle triggers pathological myocardial remodeling, leading to ventricular dilation. Dilation, on the other hand, increases the distance between the papillary muscles and the mitral annulus. The displacement of the subvalvular apparatus toward the apex of the heart results in systolic tethering of the mitral leaflets, reducing the coaptation zone [6].

Left ventricular dilation increases volume, which affects left ventricular afterload. Rising end-diastolic volume leads to a greater wall tension, as described by Laplace’s law, creating a self-perpetuating mechanism of disease progression [7].

The prevalence of IMR among patients with ischemic heart disease is estimated at approximately 15% [8,9], while it is present in about half of those with heart failure [8]. Accurate prevalence estimation is challenging due to the absence of a universally accepted definition of the disease’s dynamic nature. Multiple factors, including delayed post-infarction left ventricular remodeling, influence its progression. The defect changes over time. The use of drugs that reduce the symptoms of heart failure has a significant impact. The entire dynamics of the defect affect the delays in its diagnosis [2,3,4,10,11].

IMR secondary to coronary artery disease significantly worsens the prognosis. For patients with ischemic heart disease managed conservatively and without mitral dysfunction, the 4-year survival rate is 90%. In contrast, this rate drops to 40% for those with significant regurgitation [8,12]. The SAVE study reports nearly 30% cardiovascular mortality within three years following myocardial infarction complicated by ischemic mitral regurgitation [13]. In comparison, mortality in the same patient group without IMR was more than twice as low. Other studies report an annual mortality rate of 6% in patients without IMR, compared to 10% to 40% in those with IMR, depending on the severity of regurgitation. The above data show that the mere presence of the defect significantly affects the prognosis. For this reason, it is so important to determine the appropriate moment at which to implement the operation. In accordance with the changing guidelines, the decision to operate is postponed until severe regurgitation is diagnosed.

## 2. Objective

The primary objective was to evaluate the early and late outcomes of surgical treatment for mitral regurgitation in patients undergoing surgery for ischemic heart disease. The secondary objective was to examine the relationship between these outcomes and preoperative echocardiographic data, preoperative risk factors, the patient’s preoperative condition, and the impact of the postoperative course on late outcomes.

## 3. Materials and Methods

The analysis included 486 patients who underwent simultaneous mitral valve surgery and coronary artery bypass grafting. Exclusion criteria in the study included the following:

- Emergency surgery;
- Non-ischemic etiology of the defect;
- Intraoperative conversion of valve repair to replacement.

**Figure 1:**
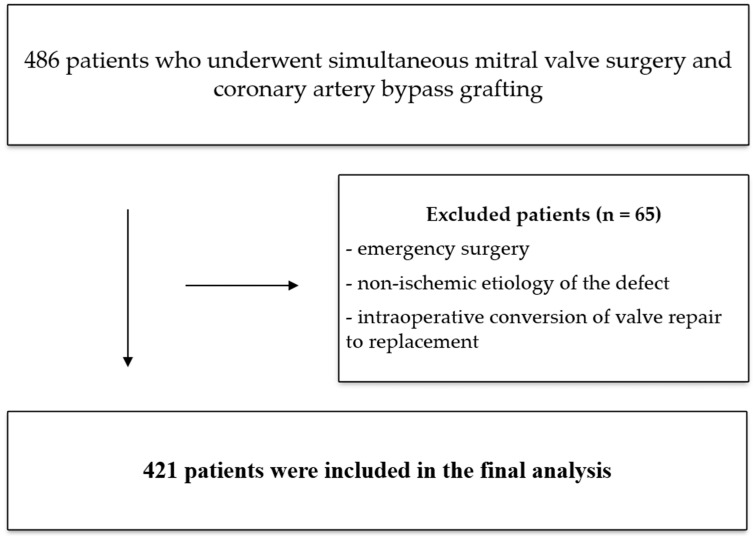
Flowchart of the study group.

The study retrospectively evaluated 421 (Figure 1) patients who underwent mitral valve repair or replacement combined with concurrent coronary artery bypass grafting (CABG). Due to the natural history of the condition, additional tricuspid valve repair was necessary for some patients during the surgery.

This study received a waiver from the Bioethical Committee of the Pomeranian Medical University because of its retrospective and observational nature (decision no. KB-0012/351/09/16 dated 29 September 2016).

Patients were admitted to the clinic one day before the planned surgery. On the day of admission, a medical history was collected, echocardiographic examinations performed outside the clinic were verified, and routine preoperative laboratory tests were conducted. Upon admission, the risk of surgery-related mortality was assessed using the standardized EuroScore (European System for Cardiac Operative Risk Evaluation).

Preoperative echocardiographic data included left ventricular ejection fraction (EF) and dimensions of the left ventricle and left atrium. Subsequent echocardiographic evaluations were routinely conducted on the first, third, and fifth postoperative days to assess changes in EF. For the purposes of this study, left atrial enlargement was classified as follows:

- Normal: <41 mm;
- Enlargement I: 41–46 mm;
- Enlargement II: 47–51 mm;
- Enlargement III: >51 mm.

At the study center, significant ischemic mitral regurgitation was treated via restrictive annuloplasty or mitral valve replacement combined with concurrent CABG. In cases of significant tricuspid regurgitation secondary to mitral valve disease, tricuspid valve repair was also performed. Surgical outcomes were standardized, as the procedures were consistently performed by the same two cardiac surgeons.

The endpoints included the following:

1. Early mortality, defined as death within the first 30 days following cardiac surgery.
2. Late mortality, defined as any death occurring more than 30 days after surgery.
3. Follow-up period, defined as the number of days survived by the patient from the 30th day post-surgery onward. The data were verified in the national registry of cardiosurgical operations.

## 4. Statistical Analysis

The statistical analysis was performed using the licensed software Statistica 13.0 (StatSoft, Inc., Tulsa, OK, USA). The group characteristics were primarily presented using means, frequencies, and percentages. The normality of variable distributions was assessed using the Shapiro–Wilk test, and variance homogeneity was tested with Levene’s test. Differences between two groups were analyzed using the Mann–Whitney U test, while differences among more than two groups were analyzed using the Kruskal–Wallis test. Qualitative data were analyzed using the Chi-squared test, with Yates’ correction applied when subgroup sample sizes were small. Associations between selected parameters were evaluated using multivariate logistic regression (adjusted by age, comorbidities, and sex) The model of regression was presented using the standard deviation and confidence interval. Survival probabilities in selected groups were presented using Kaplan–Meier curves. The log-rank test was applied to assess the Kaplan–Meier model. A significance level of *p* < 0.05 was adopted.

## 5. Results

Table 1 presents preoperative data based on the occurrence of death. In individuals over 65 years of age, a higher 30-day mortality rate was observed (*p* < 0.001). Moreover, an increased 30-day mortality was noted among patients with CCS class III (*p* = 0.007) and CCS class IV (*p* < 0.001). Statistically significant differences in mortality beyond 30 days were observed in patients with NYHA class I (*p* = 0.031), arterial hypertension (*p* = 0.003), and COPD (*p* = 0.016). Atrial fibrillation was found in 35.4% of patients upon hospital admission, and diabetes in 30.2%. However, these conditions had no significant impact on either the early or long-term outcomes of surgical treatment.

The echocardiographic data, depending on the occurrence of death after cardiac surgery, are presented in Table 2. The lowest mortality rate was observed in patients with an ejection fraction above 50% (*p* = 0.014). No other statistically significant differences were found.

**Figure 2:**
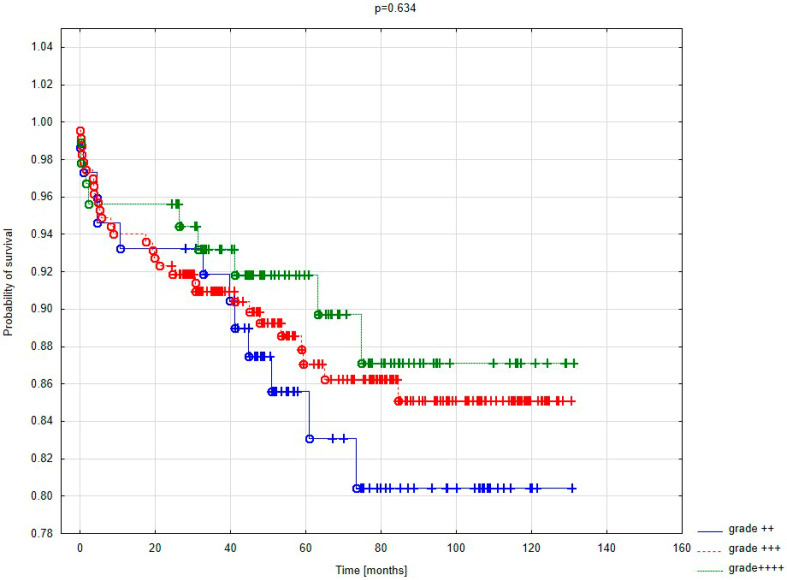
Kaplan–Meier survival curve based on the severity of regurgitant flow.

Figure 2 presents the survival curves of patients after surgery, depending on the preoperative severity of mitral valve regurgitation. In the analyzed groups, 20-month mortality was lowest among patients with preoperatively severe mitral regurgitation.

The long-term mortality analysis, extending beyond 130 months of follow-up, showed similar results. However, these findings were not statistically significant.

**Figure 3:**
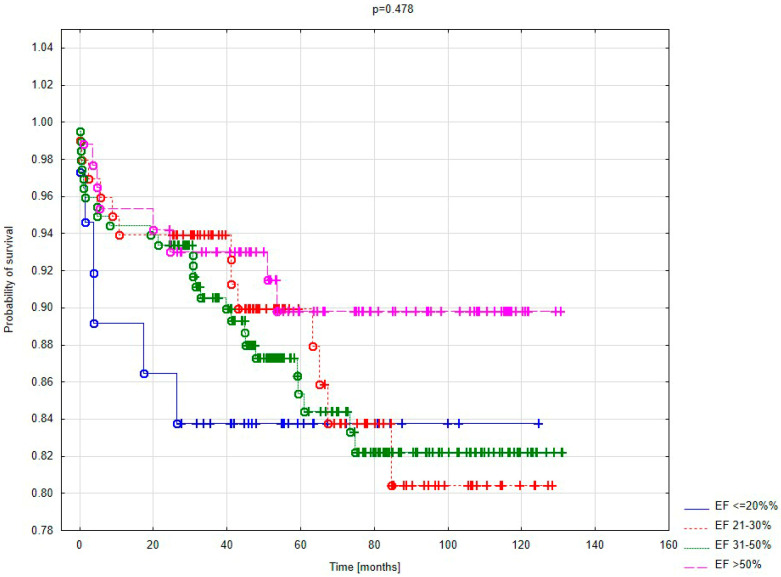
Kaplan–Meier survival curve based on EF.

Figure 3 presents survival curves based on left ventricular function, represented by ejection fraction (EF). The risk of late mortality was lowest in patients with good left ventricular function (EF > 50%). The highest mortality within the first 20 months after surgery was observed in patients with severely impaired left ventricular ejection fraction (EF ≤ 20%). However, statistical significance was not achieved.

**Figure 4:**
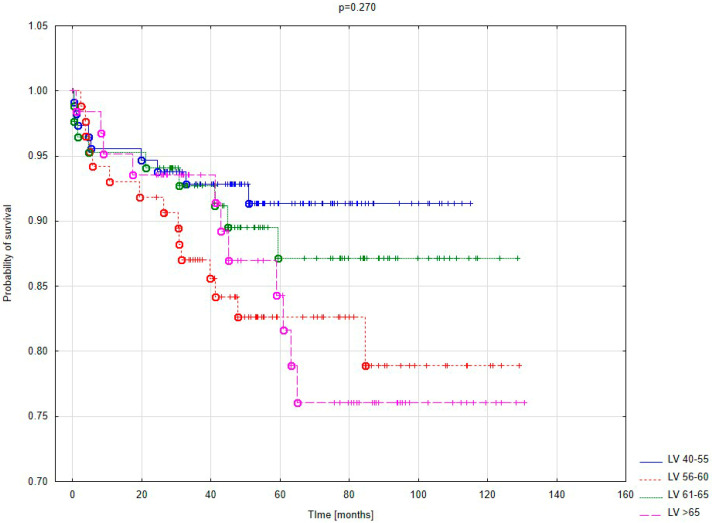
Kaplan–Meier survival curve based on LV size.

The Kaplan–Meier survival curve according to the size of the left ventricle is presented in Figure 4. No statistically significant differences were found between the survival curves. However, there is a tendency toward the best long-term outcomes in patients with normal left ventricular (LV) dimensions. Regardless of the degree of LV dilation, the results of ischemic mitral regurgitation (IMR) treatment at 20 months after surgery were nearly identical. Increased mortality among patients with severely enlarged LV becomes particularly noticeable after 60 months of follow-up.

**Figure 5:**
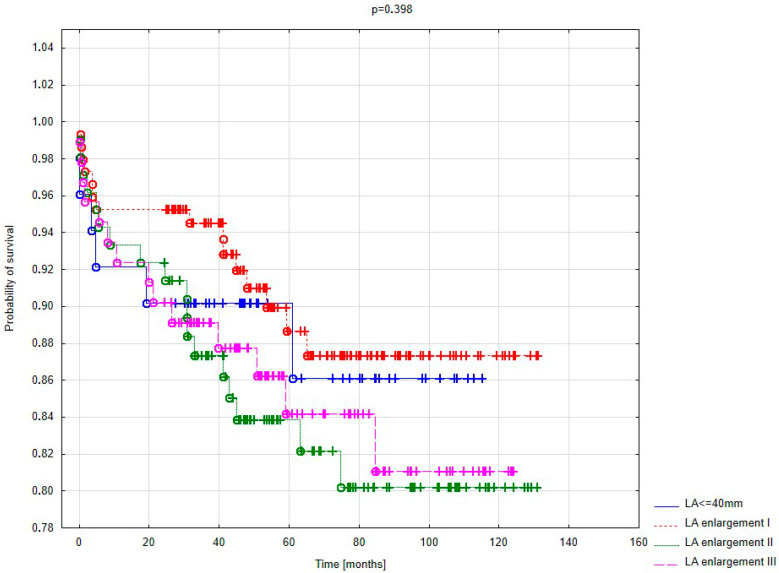
Kaplan–Meier survival curve based on the degree of LA enlargement.

Figure 5 presents the Kaplan–Meier survival curve according to left atrial size. The probability of survival was higher in patients with a non-enlarged left atrium; however, statistical significance was not achieved.

Table 3 presents an analysis of demographic data, surgical risk, and echocardiographic parameters, categorized according to left ventricular ejection fraction (LVEF) groups. Statistically significant associations across LVEF groups were found for sex (both women and men, *p* < 0.001), NYHA class II (*p* = 0.018), CCS class I (*p* = 0.002), CCS class IV (*p* = 0.009), arterial hypertension (*p* = 0.015), and atrial fibrillation (*p* = 0.010), as well as the type of mitral valve surgery, left ventricular size, and intraoperative data, including cardiopulmonary bypass time (*p* = 0.009) and aortic cross-clamping time (*p* = 0.007).

Table 4 presents an analysis of preoperative and intraoperative demographic data in relation to left ventricular size. Statistically significant associations were found with sex (*p* < 0.001); atrial fibrillation (*p* = 0.008); ejection fraction group above 50% (*p* < 0.001), 21–30 (*p* < 0.001), and below 21% (*p* < 0.006); type of valve surgery —MVP (*p* < 0.001) and MVR (*p* < 0.001); number of coronary artery bypass grafts performed (*p* = 0.018); and left atrial group below 41 mm (*p* < 0.001), 41–46 mm (*p* = 0.001), and above 51 mm (*p* < 0.001).

Table 5 presents an analysis of preoperative and intraoperative demographic data in relation to left atrial size. Statistically significant associations were found with sex (*p* = 0.005), NYHA class I (*p* < 0.001) and III (*p* < 0.001), CCS class I (*p* = 0.002) and IV (*p* = 0.003), atrial fibrillation (*p* < 0.001), type of valve surgery (*p* < 0.001), number of coronary artery bypass grafts performed (*p* = 0.006), cardiopulmonary bypass time (*p* < 0.001), and left ventricular sizes 40–50 mm (*p* < 0.001) and above 65 mm (*p* < 0.001).

The logistic regression analysis did not show any statistically significant relationship between an enlarged left atrium and the occurrence of postoperative complications. The results are presented in Table 6.

The logistic regression analysis did not show any statistically significant associations between significant and severe regurgitant flow into the left atrium and the occurrence of postoperative complications. The results are presented in Table 7.

Table 8 presents age, EuroSCORE II, and postoperative ejection fraction depending on the preoperative ejection fraction. The analysis showed significant differences between the groups based on preoperative ejection fraction and EuroSCORE II (*p* < 0.001), postoperative EF (*p* < 0.001), and delta EF (*p* < 0.001).

The association between the occurrence of complications and early mortality was assessed using logistic regression. Multivariate regression (adjusted for age, comorbidities, NYHA class, and CCS class) demonstrated an association between mortality and cardiac arrest (OR = 16.348, *p* < 0.001), ventricular fibrillation (OR = 27.725 *p* = 0.003), bradycardia (OR = 3.031, *p* = 0.050), low cardiac output syndrome (OR = 14.235, *p* < 0.001), intra-aortic balloon pump (OR = 31.022, *p* < 0.001), and reoperation (OR = 3.169, *p* = 0.011).

## 6. Discussion

The mean age of the study group was 65.7 years. It was demonstrated that age above 65 years significantly increased early mortality (*p* < 0.001). This relationship was not observed in the analysis of late mortality; however, the mean follow-up duration for patients over 65 years was 7 months shorter. This suggests that age alone may not be the sole determinant of mortality in surgical treatment of IMR.

Men accounted for nearly two-thirds of the study group. Notably, women represented 34.9% of the cohort, a proportion that significantly exceeds the 15% in the STICH trial [14]. A larger female population is associated with a cumulative higher risk of surgery. This is because women are burdened with a higher risk of surgery expressed on the EuroScore II scale.

The analysis of echocardiographic data revealed that left ventricular ejection fraction (EF) and left ventricular size were the primary factors contributing to lower mortality in women. Extremely low EF (<21%) was observed in 8.8% of men and only 2.1% of women. Severe left ventricular dilation (>65 mm) occurred in 25.8% of men and 4.8% of women. The lower prevalence of women in these extreme groups may explain the observed differences in both early and late outcomes. The assessment of EF is highly subjective. Therefore, comparing populations in terms of EF seems to be difficult. Nevertheless, the large population of patients with extremely low EF indicates the experience of the center.

The subjective severity of symptoms assessed using NYHA and CCS scales correlated with an increased early mortality only in patients classified as grade IV.

In the study group, the majority (47%) had mildly impaired left ventricular function, reflected in a reduced EF. Among the study population, 20.5% had no myocardial contractility dysfunction, with a mean EF of 59.3%. Patients at particular risk were those with an EF below 30%, constituting 32.5% of the operated group. Across the entire study group, the mean EF was 39.7%. Although this indicates moderate ventricular dysfunction, it is lower than the EF reported in other studies [15,16,17].

The data presented highlight significant EF changes after combined mitral valve and CABG surgery. The only group experiencing a postoperative EF decline comprised patients with initially good left ventricular function. Patients with low (+8.58%) and very low (+4.74%) preoperative EF demonstrated the greatest improvement. This finding suggests that patients with low EF benefit most from surgery.

No statistically significant differences in postoperative complications were found based on EF. However, low EF predisposed patients to severe complications, such as low cardiac output syndrome. Patients with a low preoperative EF were also more likely to require circulatory support using intra-aortic balloon counterpulsation (14.8% vs. 6.9%). Severe postoperative complications and an impaired left ventricular contractility directly influenced early mortality outcomes, which were significantly lower (2.3%) among patients with normal myocardial contractility. This correlation was not observed in late mortality. Although patients with extremely low EF experienced higher late mortality, the difference compared to those with an EF above 50% was not statistically significant. This indicates that while cardiac function impacts perioperative mortality, its influence on late outcomes diminishes. These results align with findings from other authors [3,10].

The severity of regurgitation did not correlate with the incidence of postoperative complications. The small number of complications in the study group may have affected this assessment. While low EF directly influenced 30-day mortality, no such association was observed with regurgitation severity. Mortality rates of 8.1% for grade 2+ regurgitation and 9.9% for grade 4+ regurgitation showed no significant statistical differences and did not exceed the operational risk estimated by EuroScore II for these groups (10.4%). In the largest group, comprising 234 patients with significant grade 3+ regurgitation, mortality exceeded the estimated operational risk (11.5%) but did not show statistically significant differences compared to other groups. Higher early mortality in this group suggests the need for a larger-scale analysis of patients with moderate and severe regurgitation. Deja’s study also found no effect of regurgitation severity on early mortality [14]. Despite the group sizes, the results in my study correspond to those of a multicenter population.

Extending the analysis of regurgitation severity on early and late complications, multivariate logistic regression analysis revealed a significantly higher odds ratio for postoperative bradycardia in patients with moderate to severe regurgitation (OR = 3.286). Patients with severe mitral regurgitation demonstrated an increased likelihood of early mortality (OR = 1.412). However, no direct relationship was documented between regurgitation severity and late mortality.

Referencing the STICH trial, it is suspected that preoperative regurgitation severity may not significantly influence late mortality [14]. Deja identified significant differences between groups with absent or mild regurgitation and those with moderate to severe regurgitation. However, differences in late mortality between patients with grade 2+ and 3+ regurgitation were not significant (44% vs. 50%). In a 6-year follow-up, Deja observed an increased mortality risk with grade 2+ (OR = 1.54) and grade 3+ regurgitation (OR = 2.01). The odds ratios for late mortality in this study are notably higher. In our study, cumulative mortality (early and late) in patients with grade 2+ regurgitation was 24% over a 6.25-year follow-up. Interestingly, mortality decreased with increasing regurgitation severity: 22.6% for grade 3+ and 19.8% for grade 4+ over slightly shorter follow-up periods (5.9 years). More detailed data analysis and differentiation of patient groups according to EF make comparison difficult. Despite this, the trend in the results in both studies is similar.

Comorbidities such as hypertension, stroke, and chronic kidney disease increased early mortality risk. Acute kidney failure or the exacerbation of chronic kidney disease, defined as a 30% rise in baseline creatinine levels, heightened 30-day mortality. Among these comorbidities, only hypertension and chronic obstructive pulmonary disease (COPD) significantly influenced late mortality. This may be due to the complex effects of persistently high blood pressure or small subgroup sizes in long-term observations of patients with chronic kidney disease and stroke. Noteworthy is the annual mortality in late follow-up observations. Among patients with COPD, stroke, and chronic kidney disease, annual mortality was approximately 6%, double that of other analyzed comorbidities.

No effect of ischemic mitral regurgitation severity on early outcomes was demonstrated. Neither regurgitant flow into the left atrium nor its size influenced early complications or 30-day mortality. However, reduced left ventricular EF significantly impacted early mortality. The difference in early mortality between patients with severely reduced EF (<21%) and those with normal EF (>50%) was nearly fivefold. Early mortality of 10.8% among patients with extreme left ventricular dysfunction correlated with the frequency of sudden cardiac arrest in this group. This suggests that sudden cardiac arrest was a direct cause of death.

The in-depth early mortality analysis utilized logistic regression (Table 9). Sudden cardiac arrest, ventricular fibrillation, low cardiac output syndrome, and intra-aortic balloon counterpulsation were associated with significantly higher mortality and were often direct causes of death. Postoperative ventricular fibrillation increased the risk of death by over 48 times (OR = 27.725). Bradycardia, another rhythm disturbance, also significantly impacted mortality, with an early mortality risk ratio of 3.031.

Another notable finding is the mortality among patients requiring reoperation for excessive bleeding. Reopening the chest tripled the risk of death (OR = 3.169). Knapik’s study similarly demonstrated that mediastinal revision significantly increases in-hospital mortality, with operational risk scales underestimating this risk [18]. However, Knapik’s study focused on isolated CABG procedures performed on cardiopulmonary bypass, which inherently carries a lower risk of postoperative bleeding.

The long-term follow-up revealed higher survival rates among patients with normal left atrial dimensions. Differences favoring smaller atrial size became evident after 60 months post-surgery. In the first three years of observation, mortality was markedly lower in patients with mildly enlarged left atria. A detailed analysis dividing patients by the degree of atrial enlargement revealed no statistically significant differences in long-term survival but a clear trend toward lower mortality in patients with normal or mildly enlarged atria. Cardiovascular complications, particularly thromboembolic events, may play a significant role in patients with markedly enlarged left atria.

Long-term survival analysis showed significantly higher survival rates among patients with normal left ventricular EF compared to those with severely reduced EF. The highest mortality was observed in patients with an EF below 21% during the first 20 months of follow-up. Differences in late survival among other EF groups became apparent only after more than 60 months of follow-up.

Left ventricular EF is widely regarded as one of the strongest prognostic factors for early and late mortality. Based on CHARM (Candesartan in Heart failure: Assessment of Reduction in Mortality and morbidity) program results, an EF up to 45% significantly impacts late mortality [19]. Findings from this study support this hypothesis. The CHARM program demonstrated the increased risks of myocardial infarction and ischemic stroke in patients with an EF below 45%. The study showed that each 10% decrease in EF increased mortality risk by 39% [19]. In this study, combining risk factors such as reduced EF and risks associated with chronic use of antiplatelet agents and vitamin K antagonists (VKA) may decisively affect survival in patients undergoing CABG and simultaneous mitral valve surgery.

The late mortality analysis by ischemic mitral regurgitation severity yielded results differing from the STICH trial. No significant differences in survival were observed up to 40 months postoperatively. Further analysis revealed that patients with severe and moderate regurgitation derive the greatest benefit from surgery. After three years, a clear trend favoring surgery qualification for patients with grade 3+ and 4+ regurgitation emerged. A prospective study evaluating valve function and coronary graft performance is necessary for detailed conclusions.

Discrepancies in long-term survival became evident after approximately 60 months, both in analyses by EF, atrial size, and regurgitation severity. This suggests the presence of other factors significantly influencing mortality. Cardiological control in the late postoperative period was most often performed outside our center. Therefore, there is no data on the drugs used, new diseases, and other factors that could affect the long-term results of the surgery.

Our study has several important limitations that should be considered when interpreting the results. First, the analysis was performed retrospectively and is based on data from a single center, which may limit the generalizability of the results to other populations. Second, although the EuroSCORE II is a widely used tool for assessing perioperative risk in cardiac surgery, its calibration and predictive validity have not been formally verified in our study population.

## 7. Conclusions

1. The degree of regurgitant flow into the LA did not significantly impact early or late outcomes of combined surgical treatment for IMR. Similarly, no effect of preoperative LA or LV size on these outcomes was observed.
2. Among the parameters measured in preoperative echocardiographic examinations, a reduced EF was associated with higher late mortality.
3. The cumulative annual risk of death in patients undergoing surgical treatment for IMR during long-term follow-up was lower than the annual mortality rates for CAD and IMR reported in the literature.

## Figures and Tables

**Table 1:** Preoperative characteristics of patients and their impact on outcomes.

	EARLY OUTCOME						LATE OUTCOME					
	Number of Patients	% of Patients	EuroScore II	Death ≤ 30 Days	% of Deaths	*p*	Liczba Chorych	Death > 30 Days	% of Deaths	% of Deaths per Year	*p*	Follow-Up (Months)
**Total**	421	100	10.0	44	10.4		377	52	13.8	2.3		73
**Women**	147	34.9	9.7	12	8.2	0.261	135	13	9.6	1.6	0.109	74
**Men**	274	65.1	10.2	32	11.7	0.261	242	39	16.1	2.7	0.109	72
**Age ≤ 65**	212	50.3	6.5	10	4.7	**<0.001**	202	23	11.4	1.8	0.345	76
**Age > 65**	209	49.7	13.6	34	16.3	**<0.001**	175	29	16.6	2.9	0.345	69
**NYHA I**	103	24.5	10.7	14	13.6	0.231	89	19	21.3	3.4	**0.031**	76
**NYHA II**	101	24.0	6.4	3	2.9	**0.008**	98	9	9.2	1.4	0.302	77
**NYHA III**	182	43.2	10.1	19	10.4	0.995	163	21	12.9	2.2	0.658	69
**NYHA IV**	35	8.3	18.1	8	22.8	**0.027**	27	3	11.1	1.9	0.659	70
**CCS I**	46	10.9	7.9	6	13.0	0.734	40	5	12.5	2.1	0.931	73
**CCS II**	109	25.9	7.4	7	6.4	0.157	102	12	11.8	1.9	0.745	74
**CCS III**	205	48.7	8.4	13	6.3	**0.007**	192	31	16.1	2.6	0.125	73
**CCS IV**	61	14.5	21.8	18	29.5	**<0.001**	43	4	9.3	1.7	0.202	67
**HA**	247	58.7	9.6	19	7.7	**0.027**	228	41	17.9	3.0	**0.003**	71
**AF**	149	35.4	9.6	17	11.4	0.634	132	24	18.2	3.2	0.083	69
**DM**	127	30.2	11.1	12	9.4	0.788	115	17	14.7	2.6	0.672	69
**CKD**	25	5.9	17.6	7	28.0	**0.002**	18	5	27.8	5.9	0.382	57
**TIA**	37	8.8	13.6	9	24.3	**0.009**	28	8	28.6	6.0	0.125	57
**COPD**	28	6.7	11.0	2	7.1	0.554	26	8	30.8	6.4	**0.016**	58

Legend: HA—arterial hypertension; DM—diabetes mellitus; NYHA—New York Heart Association; CCS—Canadian Cardiovascular Society; AF—atrial fibrillation; COPD—chronic obstructive pulmonary disease; *p*—statistical significance. Bolds and background colours are used to highlight statistically significant data, same below.

**Table 2:** Characteristics of preoperative echocardiographic findings and their impact on surgical treatment outcomes for IMR.

	Number of Patients	% of Patients	EuroScore II	Death ≤ 30 Days	% of Deaths	*p*	Number of Patients	Death > 30 Days	% of Deaths	% of Deaths per Year	*p*	Follow-Up MC.
**Total**	**421**	**100**	**10.0**	**44**	**10.4**		**377**	**49**	**13.0**	**2.3**		**73**
**IMR**	**399 ^A^**	**94.8**										
++	74	18.6 ^B^	8.8	6	8.1	0.588	68	12	17.6	1.9	0.189	75
+++	234	58.6 ^B^	9.9	27	11.5	0.536	207	26	12.6	4.2	0.737	75
++++	91	22.8 ^B^	10.9	9	9.9	0.976	82	9	11.0	1.5	0.652	71
**EF** (%)	**419 ^A^**	**99.5**										
>50	86	20.5 ^B^	6.0	2	2.3	**0.014**	84	8	9.5	1.2	0.425	82
50–31	197	47.0 ^B^	8.7	22	11.2	0.568	175	26	14.9	4.3	0.645	72
21–30	99	23.6 ^B^	13.6	14	14.1	0.199	85	13	15.3	2.2	0.803	70
<21	37	8.9 ^B^	15.4	4	10.8	0.905	33	5	15.1	1.0	0.962	60
**LV** (mm)	**381 ^A^**	**90.5**										
40–55	124	32.5 ^B^	10.4	11	8.9	0.882	113	9	7.9	1.6	0.054	68
56–60	90	23.7 ^B^	9.8	4	4.4	0.116	86	15	17.4	2.6	0.153	70
61–65	95	24.9 ^B^	8.8	10	10.5	0.602	85	11	12.9	1.8	0.796	72
>65	72	18.9 ^B^	8.9	10	13.9	0.125	62	12	19.3	1.9	0.215	75
**LA** (mm)	**396 ^A^**	**94.0**										
<41	51	12.9 ^B^	9.7	4	7.8	0.891	47	4	8.5	0.7	0.271	72
41–46	148	37.4 ^B^	9.7	12	8.1	0.514	136	15	11.0	2.5	0.319	72
47–51	105	26.5 ^B^	10.0	8	7.6	0.603	97	18	18.5	3.0	0.104	73
>51	92	23.2 ^B^	8.5	13	14.1	0.072	79	13	16.4	2.2	0.620	70

Legend: IMR—ischemic mitral regurgitation; EF—ejection fraction; LA—left atrium; LV—left ventricle; *p*—statistical significance. A—Number of results available from the entire study group of 421 patients; B—Percentage of results consistent with the size of subgroup A treated as 100%.

**Table 3:** Assessment of selected parameters in relation to EF size.

	TOTAL	EF >50%		EF 50–31%		EF 30–21%		EF <21%		*p*
		*n*	% ^B^	*n*	% ^B^	*n*	% ^B^	*n*	% ^B^	
Total	419	86	20.5	197	47.0	99	23.7	37	8.8	
Women	146	50	34.2	74	50.7	19	13.0	3	2.1	**<0.001**
Men	273	36	13.2	123	45.1	80	29.3	34	12.4	**<0.001**
NYNA I	101	14	13.9	50	49.5	29	28.7	8	7.9	0.201
NYHA II	101	28	27.7	52	51.5	17	16.8	4	4.0	**0.018**
NYHA III	182	39	21.4	80	44.0	45	24.7	18	9.9	0.719
NYHA IV	35	5	14.5	15	42.9	8	22.6	7	20.0	0.099
CCS I	46	18	39.1	22	47.8	5	10.9	1	2.2	**0.002**
CCS II	109	30	27.5	49	44.9	22	20.3	8	7.3	0.190
CCS III	205	33	16.1	101	49.3	52	25.4	19	9.2	0.182
CCS IV	59	5	8.5	25	42.4	20	33.8	9	15.3	**0.009**
HA	245	58	23.7	121	49.3	45	18.4	21	8.6	**0.015**
AF	149	38	25.5	77	51.7	27	18.1	7	4.7	**0.010**
DM	127	20	15.7	64	50.4	34	26.8	9	7.1	0.277
CKD	25	3	12.0	12	48.0	8	32.0	2	8.0	0.628
STR/TIA	35	8	22.9	14	40.0	11	31.4	2	5.7	0.551
COPD	20	4	20.0	10	50	4	20.0	2	10	0.709
**IMR**	**397 ^A^**									
IMR ++	74	19	25.7	32	43.2	16	21.6	7	9.5	0.700
IMR +++	232	45	19.4	110	47.4	59	25.4	18	7.8	0.577
IMR ++++	91	19	20.9	42	46.1	20	22.0	10	11.0	0.844
**LV** (mm)	**381 ^A^**									
40–55	124	43	34.7	61	49.2	15	12.1	5	4.0	**<0.001**
56–60	90	15	16.7	41	45.6	24	266	10	11.1	0.749
61–65	95	10	10.5	47	49.5	32	33.7	6	6.3	**0.019**
>65	72	6	8.3	28	38.9	25	34.7	13	18.1	**<0.001**
**LA** (mm)	**396 ^A^**									
<41	51	10	19.6	25	49.0	11	21.6	5	9.8	0.956
41–46	148	25	16.9	71	47.9	38	25.7	14	9.5	0.539
47–51	105	27	25.7	43	40.9	29	27.7	6	5.7	0.169
>51	92	20	21.7	45	48.9	17	18.5	10	10.9	0.514
MVP	342	55	16.1	164	47.9	94	27.5	29	8.5	**<0.001**
MVR	77	31	40.3	33	42.8	5	6.5	8	10.4	**<0.001**
MVP/MVR TVP	118	26	22.0	55	46.6	29	24.7	8	6.7	0.793
Number of CABG (avg.)	2.8	2.3		2.9		3.1		2.5		**<0.001**
CPB [minutes] (avg.)	89.7	84.1		90.9		88.0		96.7		**0.009**
ACC [minutes] (avg.)	63.0	60.0		64.1		61.4		66.6		**0.007**

Legend: HA—arterial hypertension; DM—diabetes mellitus; IMR—ischemic mitral regurgitation; ACC—aortic cross-clamping; CPB—cardiopulmonary bypass; EF—ejection fraction; TIA—transient ischemic attack; NYHA—New York Heart Association; CCS—Canadian Cardiovascular Society; COPD—chronic obstructive pulmonary disease; *p*—statistical significance. A—Number of results available from the entire study group of 421 patients; B—Percentage of results consistent with the size of subgroup A treated as 100%.

**Table 4:** Assessment of selected parameters in relation to left ventricle size.

	TOTAL	LV 40–55 mm		LV 56–60 mm		LV 61–65 mm		LV >65 mm		*p*
		*n*	% ^B^	*n*	% ^B^	*n*	% ^B^	*n*	% ^B^	
Total	**381 ^A^**	124	32.5	90	23.7	95	24.9	72	18.9	
Women	125	71	56.8	28	22.4	20	16	6	4.8	**<0.001**
Men	256	53	20.7	62	24.2	75	29.3	66	25.8	**<0.001**
NYNA I	87	23	26.4	28	32.2	24	27.6	12	13.8	0.083
NYHA II	96	34	35.5	20	20.8	20	20.8	22	22.9	0.439
NYHA III	168	59	35.2	35	20.8	40	23.8	34	20.2	0.564
NYHA IV	30	8	26.7	7	23.3	11	36.7	4	13.3	0.444
CCS I	43	19	44.2	8	18.6	7	16.3	9	20.9	0.251
CCS II	98	33	33.7	22	22.4	26	26.6	17	17.3	0.934
CCS III	192	55	28.6	50	26.0	48	25.0	39	20.4	0.365
CCS IV	48	17	35.4	10	20.8	14	29.2	7	14.6	0.739
HA	226	79	34.9	53	23.5	51	22.6	43	19.0	0.522
AF	138	56	40.6	29	21.0	23	16.7	30	21.7	**0.008**
DM	115	36	31.4	32	27.8	31	26.9	16	13.9	0.290
CKD	22	4	18.2	8	36.4	3	13.6	7	31.8	0.099
STR/TIA	32	11	34.4	8	25.0	7	21.9	6	18.7	0.926
COPD	16	4	25.0	8	50.0	1	6.3	3	18.7	0.644
**IMR**	**365 ^A^**									
IMR ++	70	24	34.3	16	22.8	20	28.6	10	14.3	0.712
IMR +++	210	70	33.3	56	26.7	49	23.3	35	16.7	0.280
IMR ++++	85	23	27.1	15	17.6	24	28.2	23	27.1	0.066
**EF** (%)	**381 ^A^**									
>50	74	43	58.1	15	20.3	8	10.8	8	10.8	**<0.001**
50–31	177	61	34.5	41	23.2	47	26.6	28	15.7	0.492
21–30	96	15	15.6	24	25.0	32	33.3	25	26.1	**<0.001**
<21	34	5	14.7	10	29.5	6	17.6	13	38.2	**0.006**
**LA** (mm)	**379 ^A^**									
<41	49	29	59.2	8	16.3	9	18.4	3	6.1	**<0.001**
41–46	141	49	34.8	36	25.5	44	31.2	12	8.5	**0.001**
47–51	101	26	25.7	29	28.7	25	24.8	21	20.8	0.274
>51	88	20	22.7	17	19.3	17	19.3	34	38.7	**<0.001**
MVP	313	89	28.4	81	25.9	87	27.8	56	17.9	**<0.001**
MVR	68	35	51.5	9	13.2	8	11.8	16	23.5	**<0.001**
MVP/MVR TVP	111	37	33.3	23	20.7	29	26.1	22	19.9	0.862
Number of CABG (avg.)	2.8	2.6		3.1		2.9		2.8		**0.018**
CPB [minutes] (avg.)	89.6	87.9		86.6		88.0		98.7		0.221
ACC [minutes] (avg.)	63.6	63.8		63.0		62.1		65.9		0.688

Legend: HA–arterial hypertension; DM—diabetes mellitus; IMR—ischemic mitral regurgitation; ACC—aortic cross-clamping; CPB—cardiopulmonary bypass; EF—ejection fraction; TIA—transient ischemic attack; NYHA—New York Heart Association; CCS—Canadian Cardiovascular Society; COPD—chronic obstructive pulmonary disease; *p*—statistical significance. A—Number of results available from the entire study group of 421 patients; B—Percentage of results consistent with the size of subgroup A treated as 100%.

**Table 5:** Assessment of selected parameters in relation to left atrial size.

	TOTAL	LA <41 mm		LA 41–46 mm		LA 47–51 mm		LA >51 mm		*p*
		*n*	% ^B^	*n*	% ^B^	*n*	% ^B^	*n*	% ^B^	
Total	**396 ^A^**	51	12.9	148	37.4	105	26.5	92	23.2	
Women	138	24	17.4	62	44.9	27	19.6	25	18.1	**0.005**
Men	258	27	10.5	86	33.4	78	30.2	67	25.9	**0.005**
NYNA I	90	13	14.4	46	51.2	25	27.7	6	6.7	**<0.001**
NYHA II	99	17	17.1	36	36.4	29	29.3	17	17.2	0.222
NYHA III	175	18	10.4	53	30.3	44	25.1	60	34.2	**<0.001**
NYHA IV	32	3	9.4	13	40.6	7	21.9	9	28.1	0.794
CCS I	44	1	2.3	10	22.7	15	34.1	18	40.9	**0.002**
CCS II	105	15	14.3	33	31.4	31	29.5	26	24.8	0.531
CCS III	198	25	12.6	80	40.4	47	23.7	46	23.3	0.543
CCS IV	49	10	20.4	25	51.0	12	24.5	2	4.1	**0.003**
HA	235	29	12.3	88	37.5	64	27.3	54	22.9	0.967
AF	146	4	2.7	34	23.3	42	28.8	66	45.2	**<0.001**
DM	119	16	13.4	50	42.1	27	22.7	26	21.8	0.551
CKD	22	0	0.0	9	40.9	9	40.9	4	18.2	0.166
STR/TIA	33	5	15.2	9	27.3	7	21.2	12	36.3	0.381
COPD	19	0	0.0	6	31.6	5	26.3	8	42.1	0.184
**IMR**	**380 ^A^**									
IMR ++	69	11	15.9	28	40.6	16	23.2	14	20.3	0.612
IMR +++	223	30	13.5	84	37.7	61	27.6	48	21.5	0.626
IMR ++++	88	8	9.1	27	30.7	24	27.3	29	32.9	0.099
**EF** (%)	**392 ^A^**									
>50	82	10	12.2	25	30.5	27	32.9	20	24.4	0.389
50–31	180	24	13.3	71	39.4	40	22.3	45	25.0	0.621
21–30	95	11	11.6	38	40.0	29	30.5	17	17.9	0.443
<21	35	5	14.3	14	40.0	6	17.1	10	28.6	0.599
**LV** (mm)	**378 ^A^**									
40–55	123	28	22.8	49	39.8	26	21.1	20	16.3	**<0.001**
56–60	90	8	8.9	36	40.0	29	32.2	17	18.9	0.254
61–65	95	9	9.5	44	46.3	25	26.3	17	17.9	0.135
>65	70	3	4.3	12	17.1	21	30.0	34	48.6	**<0.001**
MVP	323	48	14.9	129	39.9	88	27.3	58	17.9	**<0.001**
MVR	73	3	4.1	19	26.0	17	23.3	34	46.6	**<0.001**
MVP/MVR TVP	116	5	4.3	27	23.3	39	33.6	45	38.8	**<0.001**
Number of CABG (avg.)	2.8	3.1		2.9		2.7		2.5		**0.006**
CPB [minutes] (avg.)	89.3	85.3		86.4		89.0		96.7		**<0.001**
ACC [minutes] (avg.)	63.3	62.9		61.5		63.2		66.6		0.221

Legend: HA—arterial hypertension; DM—diabetes mellitus; IMR—ischemic mitral regurgitation; ACC—aortic cross-clamping; CPB—cardiopulmonary bypass; EF—ejection fraction; TIA—transient ischemic attack; NYHA—New York Heart Association; CCS—Canadian Cardiovascular Society; COPD—chronic obstructive pulmonary disease; *p*—statistical significance. A—Number of results available from the entire study group of 421 patients; B—Percentage of results consistent with the size of subgroup A treated as 100%.

**Table 6:** Assessment of the relationship between enlarged left atrium and the occurrence of postoperative complications.

	LA > 40 mm							
	Model I				Model II			
	*p*	OR	Cl −95%	Cl +95%	*p*	OR	Cl −95%	Cl +95%
Cardiac arrest	0.720	1.475	0.176	12.375	0.534	1.994	0.227	17.513
Ventricular fibrillation	0.967	0.954	0.101	9.035	0.884	0.838	0.078	9.015
Paroxysm of atrial fibrillation	0.827	0.917	0.422	1.994	0.753	0.882	0.404	1.927
Bradycardia	0.451	2.225	0.278	17.810	0.275	3.240	0.393	26.734
Low cardiac output syndrome	0.637	0.777	0.273	2.214	0.861	0.910	0.316	2.617
Intra-aortic balloon pump	0.863	0.869	0.177	4.274	0.497	1.717	0.361	8.163
Delirium syndrome	0.522	0.726	0.273	1.935	0.284	0.569	0.203	1.595
Stroke	0.641	0.680	0.135	3.439	0.614	0.665	0.136	3.242
Reoperation due to bleeding	0.187	2.708	0.617	11.891	0.161	2.877	0.657	12.603
Early mortality	0.730	1.220	0.395	3.769	0.513	1.466	0.466	4.615
Late mortality	0.374	1.654	0.546	5.012	0.294	1.829	0.592	5.649

Legend: OR—odds ratio; Cl—confidence interval; *p*—statistical significance. Notes: Model I: univariate logistic regression. Model II: multivariate regression adjusted by age, comorbidities, NYHA class, CCS class.

**Table 7:** Assessment of the relationship between significant and severe regurgitant flow into the left atrium and the occurrence of postoperative complications.

	Grade 3+ i 4+							
	Model I				Model II			
	*p*	OR	Cl −95%	Cl +95%	*p*	OR	Cl −95%	Cl +95%
Cardiac arrest	0.865	1.143	0.245	5.329	0.953	1.051	0.205	5.390
Paroxysm of atrial fibrillation	0.843	0.935	0.480	1.820	0.666	0.858	0.429	1.718
Bradycardia	0.254	3.286	0.425	25.393	0.246	3.394	0.430	26.759
Low cardiac output syndrome	0.554	0.789	0.359	1.732	0.578	0.789	0.343	1.815
Intra-aortic balloon pump	0.752	1.225	0.348	4.320	0.617	1.392	0.381	5.086
Delirium syndrome	0.823	1.110	0.444	2.780	0.801	1.132	0.432	2.963
Stroke	0.332	0.556	0.169	1.823	0.284	0.506	0.146	1.759
Reoperation due to bleeding	0.761	1.153	0.461	2.879	0.810	1.122	0.438	2.878
Early mortality	0.455	1.412	0.572	3.485	0.449	1.443	0.559	3.728
Late mortality	0.282	0.669	0.322	1.392	0.162	0.588	0.280	1.238

Legend: OR—odds ratio; Cl—confidence interval; *p*—statistical significance. Notes: Model I: univariate logistic regression. Model II: multivariate regression adjusted by age, comorbidities, NYHA class, CCS class.

**Table 8:** Postoperative changes in EF based on baseline ejection fraction.

	Preoperative EF								*p*
	EF ≤ 20% (*n* = 37)		EF 21–30% (*n* = 99)		EF 31–50% (*n* = 197)		EF > 50% (*n* = 86)		
	M	±SD	M	±SD	M	±SD	M	±SD	
Age [years]	63.5	7.2	65.4	7.9	65.8	7.3	66.3	7.9	0.246
EuroScore II [%]	15.4	13.7	13.6	12.9	8.7	7.4	6.0	5.0	**<0.001**
Postoperative EF [%]	24.2	10.5	30.6	8.3	41.1	9.8	53.3	6.7	**<0.001**
Delta EF [%]	4.7	10.9	1.9	8.5	0.0	8.8	−5.9	7.8	**<0.001**

Legend: EF—ejection fraction; *p*—statistical significance; M–mean; SD–standard deviation.

**Table 9:** Assessment of the relationship between early mortality and selected complications.

	Early Mortality							
	Model I				Model II			
	*p*	OR	Cl −95%	Cl +95%	*p*	OR	Cl −95%	Cl +95%
Cardiac arrest	**<0.001**	18.186	6.230	53.091	**<0.001**	16.348	5.634	49.287
Ventricular fibrillation	**<0.001**	48.205	5.492	423.142	**0.003**	27.725	3.040	271.410
Paroxysm of atrial fibrillation	0.290	0.593	0.225	1.561	0.415	0.662	0.246	1.785
Bradycardia	**0.038**	3.094	1.067	8.972	**0.050**	3.031	1.001	9.180
Low cardiac output syndrome	**<0.001**	13.429	6.525	27.638	**<0.001**	14.235	6.631	30.556
Intra-aortic balloon pump	**<0.001**	42.514	14.508	124.585	**<0.001**	31.022	9.530	100.983
Stroke	0.635	1.448	0.313	6.693	0.195	2.902	0.578	14.565
Reoperation due to bleeding	**0.014**	2.772	1.224	6.277	**0.011**	3.169	1.308	7.676

Legend: OR—odds ratio; Cl—confidence interval; *p*—statistical significance. Notes: Model I: univariate logistic regression. Model II: multivariate regression adjusted by age, comorbidities, NYHA class, CCS class.

## Data Availability

The data can be obtained from the corresponding author upon reasonable request.
